# Process Optimisation to Control the Physico-Chemical Characteristics of Biomimetic Nanoscale Hydroxyapatites Prepared Using Wet Chemical Precipitation

**DOI:** 10.3390/ma8052297

**Published:** 2015-04-29

**Authors:** Piergiorgio Gentile, Caroline J. Wilcock, Cheryl A. Miller, Robert Moorehead, Paul V. Hatton

**Affiliations:** School of Clinical Dentistry, University of Sheffield, 19 Claremont Crescent, Sheffield S10 2TA, UK; E-Mails: mta07cw@sheffield.ac.uk (C.J.W.); c.a.miller@sheffield.ac.uk (C.A.M.); r.moorehead@sheffield.ac.uk (R.M.)

**Keywords:** bioceramics, hydroxyapatite, nanoparticles, processing, wet-precipitation

## Abstract

Hydroxyapatite nanoscale particles (nHA) were prepared by wet chemical precipitation using four different synthesis methods. Differences in physico-chemical properties including morphology, particle-size, and crystallinity were investigated following alteration of critical processing parameters. The nanoparticles were also studied using X-ray diffraction (XRD), Fourier Transform infrared spectroscopy in attenuated total reflectance mode (FTIR-ATR), and transmission electron microscopy (TEM) with energy dispersive X-ray (EDS) spectrometry. The results showed that the particles obtained were composed of nHA, with different morphologies and aspect ratios (1.5 to 4) and degrees of crystallinity (40% to 70% following calcination) depending on the different process parameters of the synthesis method used, such as temperature, ripening time and pH. This study demonstrated that relatively small adjustments to processing conditions of different wet chemical preparation methods significantly affect the morphological and chemical characteristics of nHA. For the predicable preparation of biomimetic nHA for specific applications, the selection of both production method and careful control of processing conditions are paramount.

## 1. Introduction

Hydroxyapatite (HA) is a synthetic biomaterial commonly used in bone tissue repair and augmentation on account of its recognised biocompatibility and surface active properties [1]. Its chemical composition (Ca_10_(PO_4_)_6_(OH)_2_) is comparable to the mineral component of natural bone, and associated bioactivity promotes bone tissue regeneration at the site of implantation [2]. Furthermore, newly deposited mineral in developing bone and tooth tissues is organised at the nanoscale [3], consisting of nanocrystalline HA that is measures less than 100 nm on all axes [4,5]. These observations suggest that the use of synthetic nanoscale HA (nHA) in medical devices and regenerative medicine represents a biomimetic strategy that has the potential to improve clinical performance.

It is generally held that synthetic nHA is defined as having a grain size less than 100 nm in at least one direction, and it may provide several advantages over micro- and macro-scale HA. Various studies report that ceramic biomaterials based on nano-sized HA exhibit enhanced resorbability [6,7] and greater bioactivity [8,9] than micron-sized HA (mHA). The release of calcium ions from nHA is also similar to that from biological apatite and significantly faster than that from coarser crystals. In addition, new models for nanoscale enamel and bone demineralization suggest that demineralization reactions may be inhibited when particle sizes fall into certain critical nanoscale levels [10]. Some studies have also reported that nHA possesses a significant capability of decreasing apoptotic cell death and hence improving cell proliferation and cellular activity related to bone growth [8,11]. The improved cell proliferation and differentiation may be due to the superior surface functional properties of nano-sized HA compared to its microphase counterpart; indeed nHA has higher surface area and surface roughness, resulting in better cell adhesion and cell–matrix interactions [12]. In fact, as reported by Huang *et al*. [13] in their study, the HA size influenced dramatically the osteogenic differentiation of rat bone marrow derived mesenchymal stem cells into osteoblasts. The authors observed that nHA, compared with the traditional mHA, induced: (1) a substantial increase in the transcriptional expression of the early osteoblast-related genes including core binding factor alpha l, alkaline phosphatase and the alpha l chain of type I collagen, and (2) a higher osteoinductivity, implying that nHA changed the micro-environments of cell culture, adsorbed protein, formed a neo-matrix and significantly enhanced osteogenesis.

Several procedures for preparing nanoscale HA have been identified, involving hydrothermal, sol-gel, wet-chemical, and biomimetic deposition methods [14]. These methods are based on known chemical synthesis routes, in which accuracy is considered to be a key determinant of repeatability and outcome. Careful management of parameters such as pH, reaction time, temperature, and concentration of the reactants, together with the proper selection of the precursor materials, are therefore thought to be important during HA synthesis, as these parameters and materials may affect the composition and properties of the final product. However, the processing conditions that best reproduce the scale and morphology of natural nHA have not been reported. In this study, HA nanoparticles were therefore prepared by the wet chemical precipitation from aqueous solutions according to four protocols presented in literature, and compared to data available for nHA identified in mammalian bone. It is highly likely that biological response and therefore clinical performance is influenced by the chemistry and morphology of the HA nanoparticle, so there is now a greater need to better understand the nature of the nanoscale particles produced using the most promising methodologies. The aim of this systematic study was therefore to investigate the similarities and differences in physico-chemical properties of nHA powders prepared using different methods and conditions. This research will enable the scientific and medical device communities to make progress in the development of biomimetic nHA to improve clinical performance.

## 2. Results and Discussion

### 2.1. Results

#### 2.1.1. The Influence of Processing Conditions on the Physico-chemical Characteristics of nHA before Calcination

**Figure 1:**
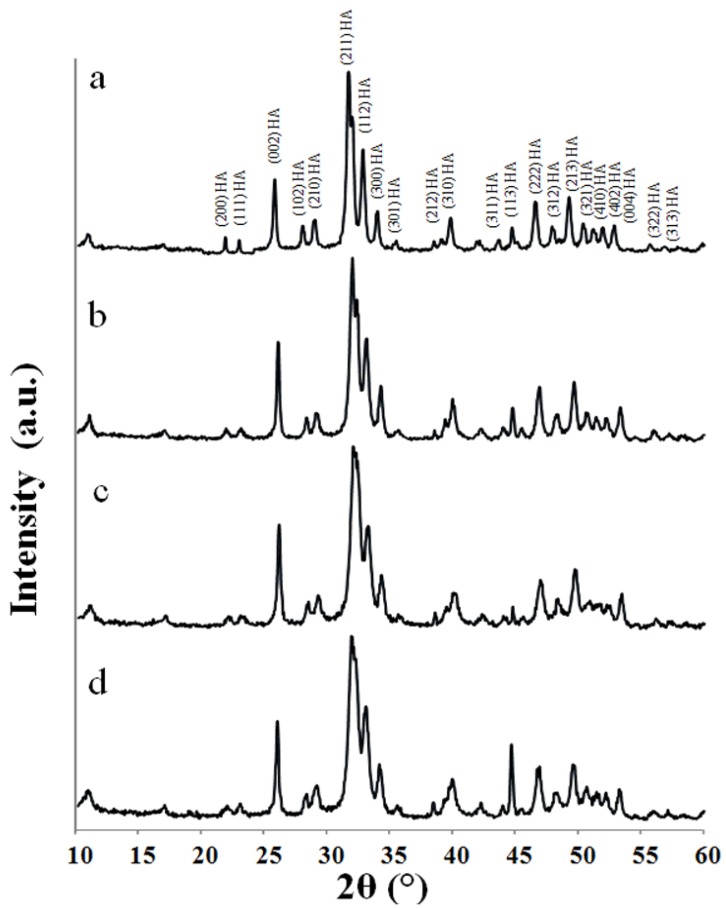
XRD patterns of as-precipitated hydroxyapatite (HA) powders synthesised following: (a) Prakash; (b) BiancoA; (c) BiancoB; and (d) Pang protocol.

XRD spectra of all the dried powders, precipitated by the four experimental methods (details given in Section 3.1), are shown in Figure 1. In all samples, only HA reflections were detected as confirmed with JCPDS file No. 09-432. Broad diffraction peaks were observed for the HA powders synthesised at low temperature (using BiancoB and Pang protocols). The increase of the synthesis temperature caused sharpened diffraction peaks, indicating increased crystallinity of nHA powders.

The observed degrees of crystallinity and crystallite size for these samples were calculated using Rietveld refinement as described in Section 3.2 and summarised in Table 1. The crystallinity ranged between 21% ± 0.9% for Pang protocol synthesised at 40 °C up to about 68% ± 0.5% for Prakash protocol prepared at 95 °C. It was noticed that the increase of the crystallinity with the temperature was not linear. There was no significant change in crystallinity when the synthetic temperature was lower than 70 °C. The crystallite sizes of the “as prepared” HA powders were less than 50 nm, and a slight increase in crystallite size of the HA powders with the increase of synthesis temperature was noted (Table 1).

**Table 1:** The χ_c_ and χ_s_ for the HA powders before and after calcination at 650 °C.

Protocols	Crystallinity χ_c_ (-)		Crystallite Size χ_s_ (nm)	
	Before calcination	After calcination	Before calcination	After calcination
*Prakash*	0.68 ± 0.05	0.70 ± 0.04	35.4 ± 2.2	25.1 ± 0.8
*BiancoA*	0.45 ± 0.08	0.61 ± 0.06	31.4 ± 1.1	28.5 ± 1.2
*BiancoB*	0.42 ± 0.09	0.47 ± 0.07	30.8 ± 1.5	29.1 ± 0.7
*Pang*	0.21 ± 0.09	0.39 ± 0.05	29.0 ± 1.9	27.0 ± 2.8

**Figure 2:**
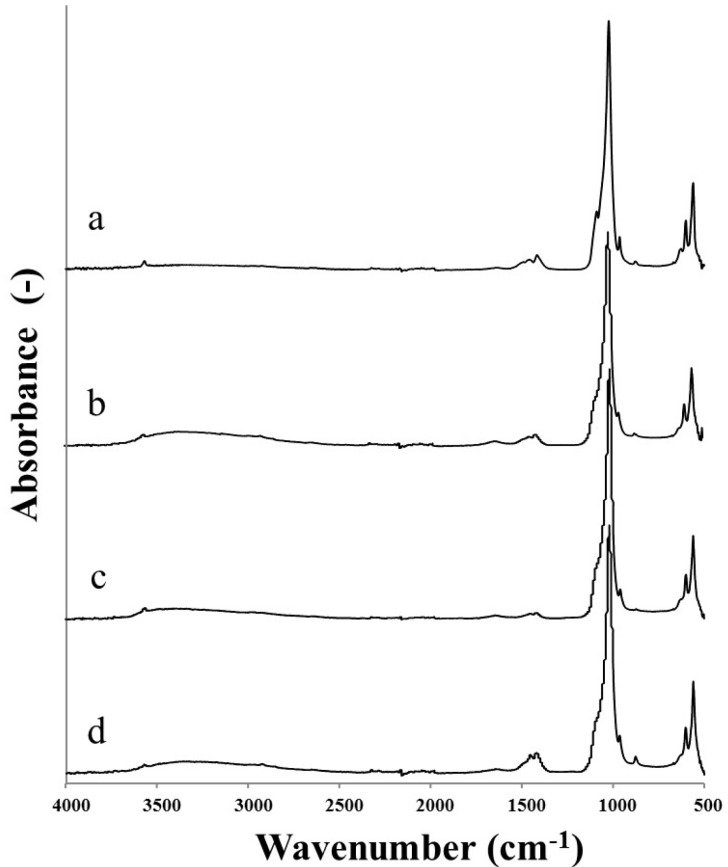
FTIR-ATR spectra of as-precipitated HA powders synthesised following: (a) Prakash; (b) BiancoA; (c) BiancoB; and (d) Pang protocol.

The FTIR-ATR analysis of all the HA powders before calcination is given in Figure 2.

The results showed that all the HA powders exhibited the characteristic bands of hydrated partially carbonated hydroxyapatite [15]: ν_OH_ (3570 cm^−^^1^) and δ_OH_ (633 cm^−^^1^); ν_1_(PO)4 (962 cm^−^^1^), ν_3_(PO)_4_ (broad band 1090–1040 cm^−^^1^) and ν_4_(PO)_4_ (565 cm^−^^1^ and 603 cm^−^^1^); ν_2_(CO)_3_ (875 cm^−^^1^) and ν_3_(CO)_3_ (1420 cm^−^^1^ and 1457 cm^−^^1^). The broad band at 3700–2500 and the sharp peak at 1637 cm^−^^1^ are most likely associated with the presence of either absorbed or combined water. The spectra contained bands attributed to carbonate substitution in HA, which probably resulted from the presence of CaCO_3_ in the reactants and dissolved CO_2_ from the atmosphere. The lack of the characteristic ν_3_(CO)_3_ peak at 1500 cm^−^^1^ suggested that only B-type carbonated hydroxyapatite was formed [16]. In the spectra obtained from the nHA produced using Prakash and BiancoB methods, the characteristic peaks (540–530 cm^−^^1^, 855 cm^−^^1^, 1130 cm^−^^1^ and 1210 cm^−^^1^) of HPO_4_ were not detected [15]. Moreover, the lack of bands in the range 750–700 indicates the absence of calcium carbonates, *i.e*., calcite (712 cm^−^^1^), aragonite (713 cm^−^^1^ and 700 cm^−^^1^) and valerite (745 cm^−^^1^). Finally, in BiancoA the absorption band at 1385 cm^−^^1^ ascribed to nitrate was also absent, due to the effective washing process.

**Figure 3:**
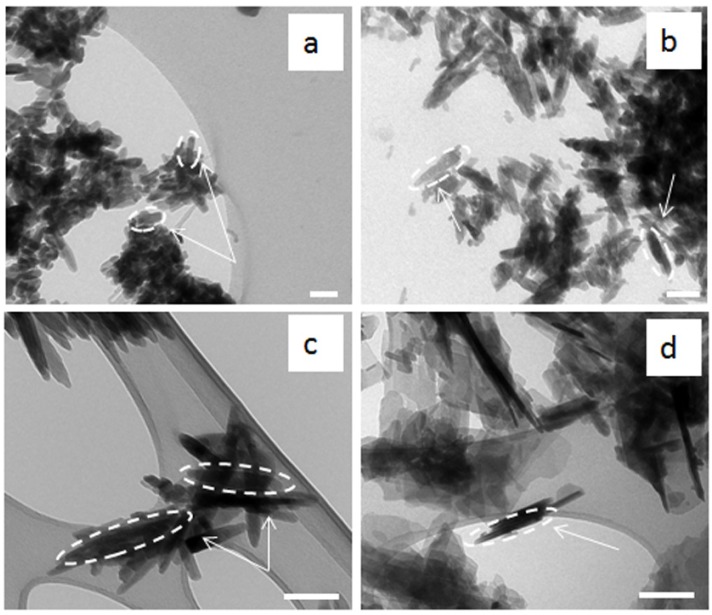
TEM micrographs of as-precipitated HA powders synthesised following: (**a**) Prakash; (**b**) BiancoA; (**c**) BiancoB; and (**d**) Pang protocol. Scale bars = 100 nm.

Figure 3 shows the TEM micrographs of the HA powders. The appearance of these nanoparticles was quite different from each other, showing nano-sized needle-like with different aspect ratios. In particular, at low synthesis temperature the HA nanoparticles carried a needle-like morphology (30–50 nm in width and 80–120 nm in length with a shape factor ranging from 3 to 4), more irregular and with less clear contours, as observed for BiancoB and Pang methods (Figure 3c,d). On the other hand, increasing the reaction temperature (up to 70 °C for the BiancoA and 95 °C for the Prakash protocol) changed the crystal from a needle-like shape to more regular form with a lower aspect ratio (20–40 nm in width and 60–80 nm in length with a shape factor about 1.5–2), with clearer contours (Figure 3a,b).

#### 2.1.2. Effect of Calcination

**Figure 4:**
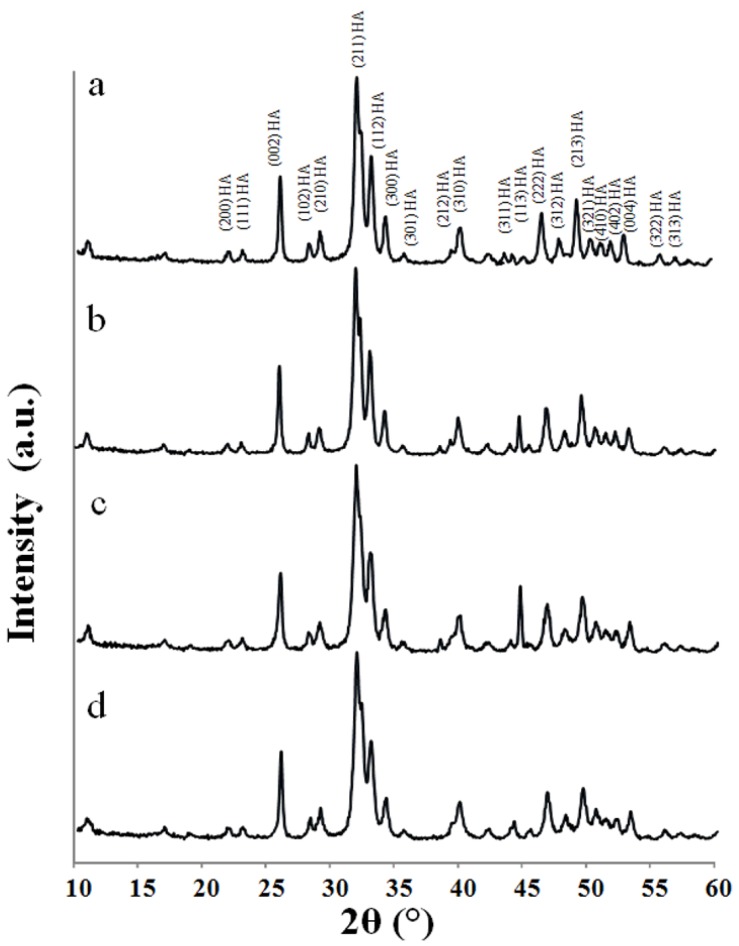
XRD patterns of calcinated HA powders synthesised following: (a) Prakash; (b) BiancoA; (c) BiancoB; and (d) Pang protocol.

The XRD diffraction patterns for the HA powders prepared following the different routes after calcination at 650 °C for 2 h are shown in Figure 4.

At 650 °C pure HA was present without any evidence of other phases of calcium phosphate or impurities. Moreover, all the calcinated HA powders showed much sharper diffraction peaks and higher crystallinity (X_c_) compared with the corresponding “as prepared” powders, as shown in Table 1. The crystallinity ranged between 39% ± 0.5% for the Pang protocol synthesised at 40 °C up to about 70% ± 0.4% for the Prakash protocol. After calcination, the *X_s_* values increase for the HA powders with lower crystallinity and smaller crystallite sizes but remain the same for those with high crystallinity and larger crystallite sizes.

**Figure 5:**
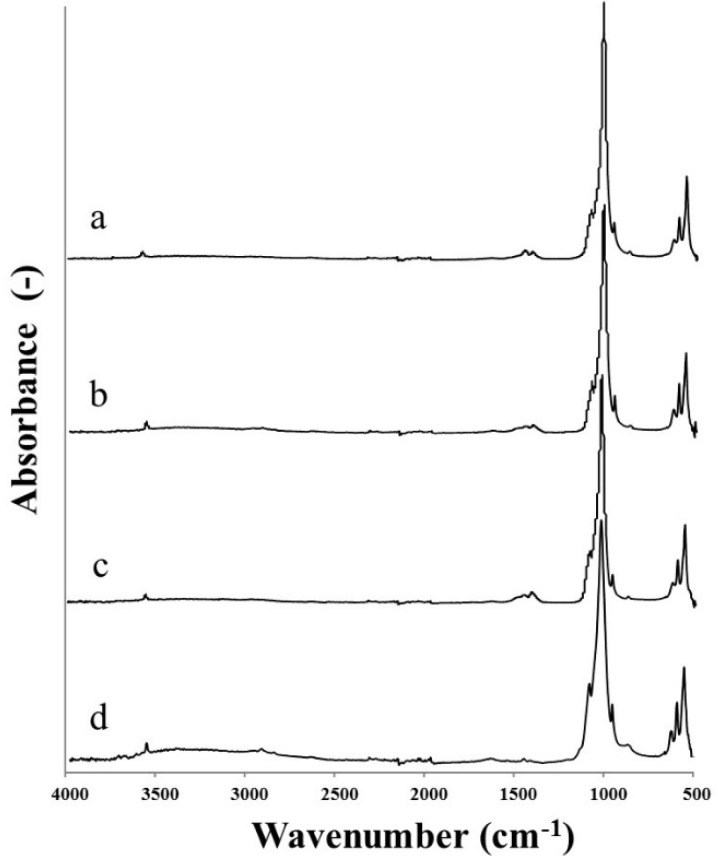
FTIR-ATR spectra of calcinated HA powders synthesised following: (a) Prakash; (b) BiancoA; (c) BiancoB; and (d) Pang protocol.

FTIR-ATR spectra of calcinated samples (Figure 5) differed considerably from those of as-precipitated materials. The intensity of peaks associated to H_2_O was noticeably decreased, indicating that the water loss had occurred during calcination.

**Figure 6:**
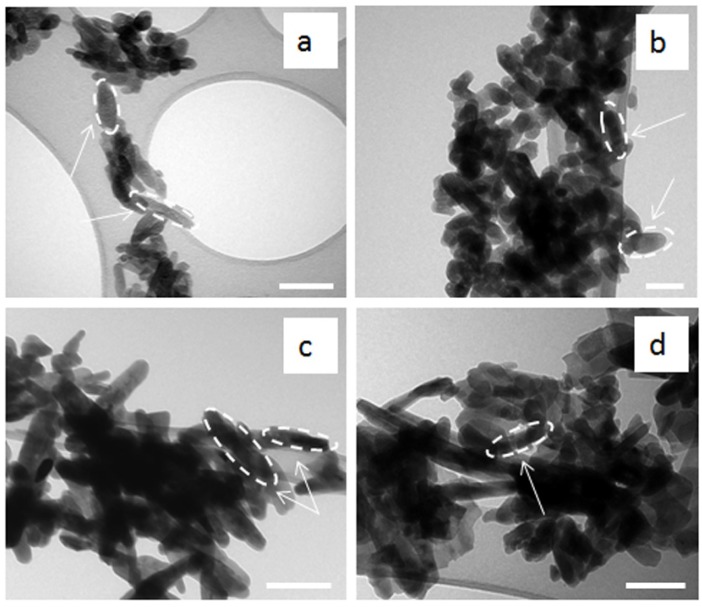
TEM micrographs of calcinated HA powders synthesised following: (**a**) Prakash; (**b**) BiancoA; (**c**) BiancoB; and (**d**) Pang protocol. Scale bars = 100 nm.

The morphology was again investigated using TEM (Figure 6). Following heat treatment, all particles showed a decrease in the size with a shape factor ranging from 1.25 to 2.5, in which a large amount of smaller new crystallites were formed during the calcination process.

Furthermore, compositional analyses were performed on all powders to evaluate the Ca to P molar ratio. EDS analysis determined that synthesised hydroxyapatite particles had a stoichiometry close to the theoretical value for apatite, ranging from 1.69 to 1.77 (Table 2) [17].

**Table 2:** The Ca/P molar ratio for the HA powders after calcination.

Protocols	Ca/P molar ratio
*Prakash*	1.69 ± 0.02
*BiancoA*	1.72 ± 0.04
*BiancoB*	1.74 ± 0.04
*Pang*	1.77 ± 0.03

### 2.2. Discussion

Wet chemical precipitation is one of the most common techniques used in the synthesis of nanoscale hydroxyapatite due to its simplicity and low cost, factors that also make it suitable for adaptation to industrial production. In particular, chemical precipitation may be accomplished using various calcium- and phosphate- containing reagents as proposed in this study. A typical procedure involves the dropwise addition of one reagent to another under continuous and gentle stirring, while the molar ratio of elements (Ca/P) is kept at stoichiometry according to its ratio in HA (1.67). As the last step, the resultant suspension may be aged under atmospheric pressure or immediately washed, filtered, dried and crushed into a powder [1,9].

In this paper, nano-hydroxyapatite powders were synthesised through wet chemical precipitation following four protocols, differing through primary reagents composition, reactant addition rate, reaction temperature, reaction pH and post-synthesis aging time. The results of this study clearly demonstrated a strong effect of the process parameters on the physico-chemical characteristics of the HA products. In particular, the crystallinity was markedly affected by the calcination temperature, reaching a value of up to approximately 70% for the HA prepared following the Prakash protocol at 95 °C. However, it was noticed that the increase of the crystallinity with temperature was not linear. In fact, a substantially increased crystallinity was observed when the synthetic temperature was greater than 70 °C, as the crystalline activation energy of the HA was overcome at that temperature. Bouyer *et al*. reported a similar phenomenon [18], in which they found that 60 °C was a transition temperature. Below that temperature the HA crystals are monocrystalline, while above this temperature the HA crystals become multicrystalline.

Further evidence for the HA crystallinity may be acquired from the FTIR-ATR spectra by evaluating the intensities of the two hydroxyl absorption bands (the sharp peak at 3570 cm^−^^1^ and 1637 cm^−^^1^) and the band at 940 cm^−^^1^ for phosphate group [19]. It was seen that the intensities of these three bands increased with the synthesis temperature. The result was consistent with the obtained XRD results. In addition, the broad band at 3700–2500 cm^−^^1^, which is a reflection of the combined water in HA powders, also decreased with increasing synthesis temperature. The lower water content resulted from the higher crystallinity of HA powder, implying the crystalline HA powder had less affinity for water than its amorphous counterpart (as observed for the nHA prepared by the Prakash protocol).

The analysis of the HA morphology made an important contribution to this study, because it has been reported previously that small crystals (nano-size) were suitable due to potentially greater bioactivity when compared to coarse crystals (submicron-size) [20]. Moreover, the finer crystals also provided larger interfaces for osseointegration [21]. The morphological behaviour observed in all the precipitated powders, in which the appearance of these nanoparticles was quite different from each other and may be related with the synthesis temperature, which effects the nano-powders crystal shape. The change from needle-like (with irregular shape and contours) to a more regular shape (lower aspect ratio) is strictly related to the increase in the synthesis temperature, corresponding with an increase of the crystallinity of the HA nano-crystals. In effect, a more regular shape of the particles was observed when the powders had higher crystallinity, as shown in Figure 6a for Prakash protocol. [22]. Furthermore, it was noticed that the increase of the synthesis temperature increased slightly the HA nanoparticle size. The exact particle nucleation and growth mechanisms are not clear. Nucleation occurs probably by either hydrolytic reactions or a salting-out phenomenon. Growth could be via diffusive molecular deposition or an aggregation of primary particles due to increased precipitation temperatures [23].

Moreover, the influence of calcination on the physico-chemical characteristics of HA nanoparticles was also investigated. The calcination temperature of 650 °C was selected to avoid the formation of impurities that could be introduced at higher temperatures (*i.e*., tricalcium phosphate content above 900 °C) [24]. The calcinated HA powders showed much higher crystallinity and sharper diffraction peaks compared with the as-precipitated ones, as confirmed by the infrared spectra, that showed a decrease in the intensity of peaks associated to H_2_O, indicating the water loss occurred during calcination. However, the small sharp peak at 3571 cm^−^^1^, attributed to the hydroxyl group of HA, remained after calcination. These results suggested that no dehydration occurred within the HA molecules during calcination, and the water loss in the samples resulted from the loss of combined water in the HA powders [24].

Furthermore, it was interesting to notice that the change in crystallite sizes after calcination was dependent on the crystallinity and crystallite sizes of “as prepared” HA powders. After calcination, the *X_s_* values increased for the HA powders with lower crystallinity and smaller crystallite sizes but remained the same for those with high crystallinity and larger crystallite sizes. This unusual change in crystallite size after calcination was also observed by Gibson *et al*. [25] for apatites after heating to temperatures between 650 °C and 750 °C for 2 h. Finally, after heat treatment at 650 °C, all the particles showed a decrease in the size with a shape factor ranging from 1.25 to 2.5. This was most likely due to a large amount of relatively smaller new crystallites forming during the calcination, as reported previously [22].

## 3. Experimental Section

### 3.1. Hydroxyapatite Nanophase Synthesis

HA powders were prepared by the direct precipitation from aqueous solutions according four different protocols. Many factors influence the HA properties since these different methods are characterised by different starting materials, pH (in a range from 6.5 to 12), temperature (ranging from 25 to 95 °C), and ripening time (from 24 to 96 h). In details:

Prakash protocol [26]. HA was precipitated using the reaction formula shown below:

(1) $${\text{10Ca(OH)}}_{2}{\text{+6H}}_{3}{\text{PO}}_{4}\to {\text{Ca}}_{10}{{\text{(PO}}_{4})}_{6}{\text{(OH)}}_{2}{\text{+18H}}_{2}\text{O}$$

Ca(OH)_2_ and 85 wt% H_3_PO_4_ (>99% pure) was purchased from Sigma-Aldrich, St. Louis, MO, USA. The quantity of various reactants used are as follows: 1.85 g of Ca(OH)_2_ powder in 250 mL of deionized (DI) water and 1.73 g of 85 wt% H_3_PO_4_ in 250 mL of DI water. The acid was added to the base at a rate approximately equal to 3.5 mL/min using a peristaltic pump, and prior to PO_4_^3^^−^ ion addition to Ca^2+^ ion solution, the latter was heated to reaction temperature at 95 °C and stirred for 1 h at 400 rpm. The temperature was maintained within ±2 °C.

BiancoA and BiancoB protocols [22]. Nanoscale HA powders were prepared under magnetic stirring (around 400 rpm) following two different precipitation routes:

(i) from calcium nitrate (coded as BiancoA),
(ii) from calcium hydroxide (coded as BiancoB).

*BiancoA Protocol*. Stoichiometric volume of calcium nitrate tetrahydrate (Ca(NO_3_)_2_·4H_2_O, Sigma-Aldrich 99.2%) aqueous solution was added dropwise to diammonium hydrogen phosphate ((NH_4_)_2_HPO_4_, Sigma-Aldrich 99.2%) aqueous solution.

The following reaction occurred:

(2) $${\text{10Ca(NO}}_{3}{)}_{2}{\text{+6(NH}}_{4}{\text{)HPO}}_{4}{\text{+2H}}_{2}\text{O}\to {\text{Ca}}_{10}{{\text{(PO}}_{4})}_{6}{\text{(OH)}}_{2}{\text{+12NH}}_{4}{\text{NO}}_{3}{\text{+8HNO}}_{3}$$

During precipitation, the pH was continuously monitored and adjusted at 10 ± 0.1 by adding NH_4_OH (Sigma-Aldrich). Precipitates were aged in situ for 24 h at 70 °C and then washed several times with NH4OH aqueous solution (pH 10).

*BiancoB Protocol*. An aqueous suspension of calcium hydroxide (Ca(OH)_2_, Sigma-Aldrich 99.5%) was titrated with phosphoric acid (H_3_PO_4_, Sigma-Aldrich 85%). The following reaction occurred as Equation (1). The pH was finally adjusted at 9.4 by adding NH_4_OH. As-precipitated powder was aged in situ for 24 h at room temperature and washed several times with NH_4_OH aqueous solution (pH 9.4).

Pang protocol [24]. HA nanoparticles were prepared by chemical precipitation through aqueous solutions of the reactants. Calcium chloride and ammonium hydrogenphosphate (both supplied by Sigma-Aldrich, St. Louis, MO, USA) were first dissolved in deionised water to form 0.5 and 0.3 M aqueous solutions, respectively. Equal amounts of these two aqueous solutions were separately pre-heated to the synthesis temperature, and then mixed under vigorous stirring at 40 °C.

(3) $$10{\text{CaCl}}_{2}+6{\text{(NH}}_{4}{\text{)HPO}}_{4}+8{\text{NH}}_{4}\text{OH}\to {\text{Ca}}_{10}{{\text{(PO}}_{4})}_{6}{\text{(OH)}}_{2}+20{\text{NH}}_{4}\text{Cl}+6{\text{H}}_{2}\text{O}$$

Meanwhile, ammonium hydroxide (Sigma-Aldrich) was added immediately to adjust the reaction mixture to pH 10. The pH value was kept constant throughout the experiment. After ripening for a specified period of time (96 h), the precipitates were recovered by centrifugation and then washed with water. At least five cycles of washing and centrifuging were required to ensure complete removal of the by-product, ammonium chloride.

All the reactants used in this study were dissolved in deionized (DI) water produced using a Milli-Q unit (Millipore, Watford, UK) and the conductivity of the DI water was 0.05 µS/cm at 25 °C.

The calcination of HA powders synthesised under different temperatures and ripening times was carried out by first drying the samples at 70 °C for 24 h in oven and then calcination at 650 °C for 6 h at the ramp rate of 5.0 °C/min with a dwell time of 2 h in a tube furnace.

### 3.2. Physical, Chemical and Morphological Characterisation

X-ray powder diffraction (XRD) studies were carried out on a Philips RW1710 powder diffractometer, using Bragg-Brentano geometry with CuKα radiation and a solid-state Peltier cooled detector. All powder diffraction spectra were measured in continuous mode using the following conditions: 2θ angular range 10°–60°, tube power 45 kV and 40 mA, 2θ step size 0.02° and a scan rate of 1° 2θ/min. The diffraction spectra were compared with those in the Powder Diffraction Files (PDF) database of the Joint Committee on Powder Diffraction Standards (JCPDS). At least five spectra were recorded for each sample, and the results were averaged with standard deviation. The crystallinity degree (*X_c_*), corresponding to the fraction of crystalline phase present in the examined volume, was evaluated as follows:

(4) $$X_{c}=1-(V_{112/300}/I_{300})$$

where I_300_ is the intensity of (300) reflection of HA and V_112/300_ is the intensity of the hollow between (112) and (300) reflections, which completely disappears in non-crystalline samples. In agreement with Landi *et al*. [27] a verification was done as follows:

(5) $$B_{002}\sqrt[{3}]{X_{c}}=K$$

where *K* is a constant found equal to 0.24 for a very large number of different HA powders, and B_002_ is the full width at half maximum (in degrees) of reflection (002).

Moreover, the peak broadening of XRD reflection can be used to estimate the crystallite size in a direction perpendicular to the crystallographic plane based on Scherrer’s formula as follows [28]:

(6) $$X_{s}=0.9\text{λ}(FWHM\cdot \cos\vartheta )$$

where *X_s_* is the crystallite size (nm), λ the wavelength of monochromatic X-ray beam (nm) (λ = 0.15406 nm for CuKα radiation); FWHM the full width at half maximum for the diffraction peak under consideration (rad); and ϑ the diffraction angle (°). The diffraction peak at 2θ = 26.04° was selected for calculation of the crystallite size since it is sharper and isolated from others. This peak assigns to (002) Miller’s plane family and shows the crystal growth along the c-axis of the HA crystalline structure [29].

FTIR-ATR spectra were obtained using a Thermoscientific Nikolett spectrometer (Unicam Ltd., Cambridge, England). The spectra were recorded between 500 and 4000 cm^–1^ with a spectral resolution of 4 cm^–1^ averaging 64 scans. The HA powders used for FTIR-ATR measurement were dried in a vacuum oven at 70 °C for 1 week before testing.

The morphology and size distribution of the synthesized HA powders was characterised with a transmission electron microscope (TEM; JEM 100CX, Jeol, Tokyo, Japan), operating at 80 kV and a particle size analyzer (ImageJ, Maryland, Bethesda, MD, USA), respectively. An aspect ratio can be defined by the ratio length/width of the HA nanocrystals:

(7) $$F_{s}=L/l$$

where *F_s_* is the shape factor; *L* is particle length (nm) and *l* is particle width (nm). At least five images were recorded for each sample.

Finally compositional analysis by EDS (Philips EDAX 9100, EDAX Inc., Mahwah, NJ, USA) was performed on all powders to evaluate the Ca to P molar ratio. Three replicates were measured, and the results were averaged with standard deviation.

## 4. Conclusions

HA nanoparticles with a stoichiometry close to the theoretical value for apatite were successfully synthesised by wet-chemical precipitation using four different protocols. Products showed different physico-chemical properties depending upon the adjustment of process parameters as well as the choice of synthesis method. In particular, the synthesis temperature influenced nanoparticle shape, with lower temperatures producing a more needle-like morphology, characterised by a shape factor of 3-4 (BiancoB and Pang). Increasing the reaction temperature caused the crystal morphology to change from a needle-like shape to a more regular form with a lower aspect ratio (1.5 for Prakash and BiancoA). Furthermore, the synthesis temperature influenced crystallinity, ranging from between 40% for Pang up to about 70% for the nHA obtained following the Prakash protocol, and confirmed by XRD and FTIR-ATR studies.

Controlling the particle morphology of this biomaterial is of utmost importance because its shape and size are known to be useful to mimic nHA in mammalian bone and tooth. Though dimensions of biological bone apatite crystals reported in the literature vary due to different treatment methods and analytical techniques, it is generally at the nanoscale with values in the ranges of 30–50 nm (length), 15–30 nm (width) with an aspect ratio ranging from 1.5 to 2. The Prakash protocol may be considered to be the most suited to mimic bone apatite and to industrial scale-up of nHA, as it is based on a relatively simple reaction involving aqueous solutions of calcium hydroxide and phosphoric acid, with only water as a by-product. However, with regards to nHA found in dental tissues, typical apatite crystals in enamel are rod-like in shape with widths of 25–100 nm and undetermined lengths of greater than 100 nm along the c-axis. Therefore, BiancoB protocol is considered to be more appropriate to produce nHA that mimics that found in tooth tissue. Moreover, nHA with different aspect ratio may be used as inorganic filler for the preparation of scaffolds for bone tissue engineering in order to enhance the mechanical properties and to improve the biological activity. Specifically nHA with low aspect ratio can be deposited onto the walls of porous freeze-dried polymeric scaffolds, while nanoparticles with high aspect ratio can be dispersed longitudinally across the polymeric fibers in electrospun membranes. In conclusion, the aqueous wet-chemical precipitation methods investigated here provide a versatile platform for preparation of different nHA biomaterials for use in devices and regenerative medicine products, but great care should be exercised when optimising process parameters to generate nHA with desired physico-chemical properties.
